# Longitudinal associations between oral health impacts and quality of life among a national cohort of Thai adults

**DOI:** 10.1186/1477-7525-11-172

**Published:** 2013-10-18

**Authors:** Vasoontara Yiengprugsawan, Tewarit Somkotra, Sam-ang Seubsman, Adrian C Sleigh

**Affiliations:** 1 National Centre for Epidemiology and Population Health, The Australian National University, Canberra, Australia; 2 Department of Community Dentistry, Faculty of Dentistry, Chulalongkorn University, Patumwan, Bangkok, Thailand; 3 School of Human Ecology, Sukhothai Thammathirat Open University, Nonthaburi, Thailand

**Keywords:** Oral health, Longitudinal analysis, Cohort study, Quality of life, Thailand

## Abstract

**Background:**

There is limited evidence on the association between oral health and general health in middle-income countries. This study analysed data from 60,569 adult students enrolled at Sukhothai Thammathirat Open University and residing throughout Thailand who reported oral health impacts at the 2005 baseline and 2009 health status based on Short Form (SF-8) survey.

**Findings:**

In 2005, 16.4% had difficulty chewing and/or swallowing, 13.4% reported difficulty speaking and/or discomfort with social interaction, and 10.8% of the cohort reported having pain associated with teeth or dentures. Cohort members reporting one or more oral health impacts in 2005 had lower SF-8 mean scores in 2009. In particular, monotonic dose–response gradients in 2005–2009 associations based on multivariate linear regression were found between an increase in number of oral impacts (0, 1, 2, 3) and a decline in SF-8 Physical Component Summary scores (adjusted means of 50.5, 49.2, 48.6, 47.9) as well as SF-8 Mental Component Summary scores (adjusted means of 43.2, 40.9, 40.3, 38.6) in younger cohort members. Similar dose response gradients were found in older cohort members.

**Conclusions:**

We found strong association between oral health impacts and adverse health and quality of life among Thai adults. This finding confirms that oral health is one of the key determinants of population health.

## Background

Oral health is accepted by the World Health Organization as a vital component of overall health and quality of life [1,2]. Oral health impacts are multifaceted and include physical dental function, pain, psychological discomfort, and social impact – all of which profoundly affect overall well-being [3-5]. Associations between oral health and overall quality of life have been noted in previous studies, mostly cross-sectional surveys of older persons in Western settings [6-9].

Less is known on oral health and general health in the adult populations of middle-income Asian countries since most studies focus on children or the elderly [10-14]. This is unfortunate as countries with emerging economies need data to plan and develop oral health care systems for appropriate population subgroups. Policy makers in transitional middle-income countries especially need local longitudinal data to confirm suspected etiological links between oral health and quality of life. This information will enable the development and justification of an appropriate mix of preventive and restorative care for populations of all ages.

Our work on oral health has been directed at these knowledge gaps. In earlier reports related to our studies of health-risk transition in Thailand, we have identified risk factors associated cross-sectionally with tooth loss, oral health, and quality of life in adults [15,16]. In other cross-sectional research on health services and equity in Thailand, we noted the social determinants of oral health among middle aged and older adults [14,17]. In the study reported here, we examine etiological association between oral health impacts, overall health, and quality of life among Thai adults.

## Methods

### Study population

This report is part of the 'Thai Health-Risk Transition: a National Cohort Study’, investigating health-risk dynamics as Thais move on from traditional illnesses (maternal and child mortality and infectious diseases) to emerging chronic diseases and injury [18]. The 20-page comprehensive baseline questionnaire on health risks and diseases was sent to 200,000 Sukhothai Thammathirat Open University (STOU) students who were enrolled in 2005 and resided throughout Thailand. The STOU President and study leaders assured the participants that their personal information will not be revealed at the individual level or have any influence on academic progress at STOU. As a result, there were 87,134 adult students who responded to the self-completed questionnaire and these constitute our cohort. The cohort represented well the Thai population for socioeconomic status (modest income levels) and geographic distribution [19]. There was a slight excess of females, and the overall median age was 29 years. A four-year follow-up was conducted in 2009 (70% response rate, n = 60,569) and the resulting 2005–2009 data were the basis of this analysis.

### Exposure, outcome, and covariates

At baseline in 2005, oral health impacts were assessed by the following questions: “Do your teeth or dentures currently cause you…: 'difficulty speaking’, 'difficulty swallowing’, 'difficulty chewing’, 'difficulty with social interaction’, and/or 'pain’. For analysis, these five oral health conditions were combined into three oral health impacts following the Oral Impacts on Daily Performances (OIDP) to capture physical, psychological and social dimensions: *physical* (difficulty chewing and/or swallowing), *psychological* (pain), and *social* (difficulty speaking and/or difficulty with social interaction) [20,21].

Health quality of life outcomes were measured using the SF-8 questionnaire (Medical Outcomes Study Short-Form 8) which includes eight domains: general health, physical functioning, role physical, bodily pain, vitality, social functioning, mental health, and role emotional. All categorical responses for each domain are numerically scaled according to the SF-8 international standard defined by the RAND Corporation [22,23]. Higher scores indicate better status. To compute summary scores, international physical or mental weights were applied to each domain value before all eight domains were summed and a physical or mental constant added [22,23]. The resulting Physical Component Summary (PCS) and Mental Component Summary (MCS) scores are designed for a normal population to have an average value of 50 and standard deviation of 10 (norm-based standardisation).

We also gathered information on an array of potential covariates or confounders that could influence the health and quality of life outcomes. Included were number of teeth at 2005 baseline, 2009 socio-demo-geographic characteristics (sex, age in years, household income, and rural vs urban residence), health-risk behaviour (smoking and alcohol drinking), and reported doctor-diagnosed chronic disorders (diabetes, hypertension, heart disease). These variables have been shown to influence various outcomes previously reported in our cohort [15,16,24].

### Data processing and statistical analysis

Data scanning and editing were done using Thai Scandevet, SQL and SPSS software. For analysis we used Stata version 12. Individuals with missing data were excluded from analyses presented here so totals vary slightly according to the information available. Analyses were stratified by age groups for two main reasons: first, cohort members consist of two thirds aged less than 40 and a third older than 40 years and our previous studies have reported substantial differences in oral health and health behaviours in these two groups [15,16]. Secondly, previous research in the cohort showed different patterns of self-assessed physical and mental health among younger and older cohort members with a decline in physical health but improvement in mental health observed in older cohort members [23,24].

In the final analyses, we use multivariate linear regressions to examine the association between baseline oral health and 4-year follow-up Physical Component Summary (PCS) and Mental Component Summary (MCS) scores. Means scores and 95% confidence intervals were adjusted for potential confounders.

### Ethical considerations

Ethics approval was obtained from Sukhothai Thammathirat Open University Research and Development Institute (protocol 0522/10) and the Australian National University Human Research Ethics Committee (protocol 2004/344 and 2009/570). Informed written consent was obtained from all participants.

## Results

Characteristics of cohort members at 4-year follow-up in 2009 are summarised in Table 1: 53.6% of respondents were aged below 40 years and 54.7% of the cohort were female. Younger respondents (aged <40 years) were almost twice as likely to report lower household monthly income of less than <7000 Baht per month. Older respondents (aged 40+ years) tended to live in urban areas, reported a much higher prevalence of chronic disorders, and were more likely to engage in smoking and alcohol drinking.

**Table 1:** Attributes of Thai cohort members in 2009

**Cohort attributes (n = 60,569)**	**Overall (%)**	**Age groups (column %)**	
		**<40 years**	**≥40 years**
**Overall**		66.6	33.4
**Socio-demo-geographic characteristics in 2009**			
Males	45.3	40.1	55.6
Females	54.7	59.9	45.4
*Household monthly income* (*Baht*)*			
<7000	11.2	13.2	7.2
7001-10000	10.0	12.0	5.9
10000-20000	23.6	27.0	16.9
20000-30000	21.3	21.5	21.0
>30000	33.9	26.3	49.0
*Current residence*			
Rural	44.0	45.8	40.4
Urban	56.0	54.2	59.6
**Health status and risk behaviours in 2009**			
*Reported doctor*-*diagnosed diseases in 2009*			
Metabolic or cardiovascular disorders	18.6	9.8	36.2
*Smoking*			
Regular smoker	8.9	8.1	10.4
Former smoker	14.3	10.4	22.2
*Alcohol drinking*			
Regular alcohol drinker	17.6	16.2	20.4
**Oral health impacts in 2005**			
Difficulties with chewing and/or swallowing	16.4	13.4	22.5
Difficulties speaking and/or discomfort with social interaction	13.4	13.5	13.2
Pain associated with teeth or dentures	10.8	11.2	10.0
*Number of oral health impacts*			
1	20.2	18.9	22.9
2	8.0	7.5	9.1
3	1.8	1.7	1.9
*Number of natural teeth*			
<20 teeth	4.2	3.0	6.6

*In 2009, 30 Baht ~ 1$US.

Oral health impacts at baseline in 2005 were as follows: 16.4% reported difficulty chewing and/or swallowing, 13.4% difficulty speaking and/or discomfort with social interaction, and 10.8% reported having pain associated with teeth or dentures. Comparing age groups, older persons were much more likely to report difficulty chewing and/or swallowing (22.5% vs 13.4%). In contrast, for the two age groups, prevalence was similar for difficulties with social setting and pain. Means and standard deviations of the eight SF-8 domains and the Physical Component Summary (PCS) and Mental Component Summary (MCS) scores are reported (Table 2). Cohort members with no oral impact had higher mean scores in all domains as well as higher physical and mental summary scores in both age groups (p < 0.005).

**Table 2:** **Oral health impacts and Short Form Medical Outcome** (**SF**-**8**) **scores**, **Thai Cohort Study**

**Age**	**Oral health impacts in 2005**	**2009 SF-8 mean scores (± standard deviations)**									
		SF-8 domain scores*								Summary scores**	
		**GH**	**PF**	**RP**	**BP**	**VT**	**SF**	**MH**	**RE**	**PCS**	**MCS**
<40	No oral health impact	44.6(±5.9)	46.4(±8.0)	48.6(±6.2)	51.8(±7.2)	51.7(±7.7)	47.8(±6.9)	45.5(±7.8)	46.5(±6.0)	49.6(±6.8)	46.8(±8.4)
	Difficulty chewing and/or swallowing	43.0(±6.0)	45.5(±7.9)	46.9(±6.6)	49.7(±7.3)	49.5(±8.2)	46.3(±7.1)	43.3(±8.4)	45.1(±6.5)	47.8(±7.2)	44.3(±9.0)
	Difficulty speaking and/or discomfort with social interaction	42.7(±6.0)	45.6(±7.8)	46.8(±6.6)	49.7(±7.4)	49.2(±8.2)	46.1(±7.2)	42.6(±8.6)	44.6(±6.6)	47.9(±7.3)	43.5(±9.1)
	Pain associated with teeth or dentures	42.7(±6.0)	45.5(±7.8)	46.6(±6.7)	49.1(±7.4)	49.4(±8.1)	46.1(±7.4)	42.7(±8.4)	44.8(±6.5)	47.6(±7.3)	43.8(±9.1)
≥40	No oral health impact	45.0(±5.7)	45.6(±8.4)	49.0(±6.2)	50.6(±7.5)	52.4(±7.5)	47.8(±7.0)	47.7(±7.0)	47.2(±5.7)	49.3(±6.9)	48.9(±7.7)
	Difficulty chewing and/or swallowing	43.4(±5.9)	44.4(±8.2)	47.2(±6.8)	50.2(±7.6)	50.4(±7.8)	46.4(±7.0)	45.8(±7.5)	45.8(±6.2)	47.0(±6.9)	46.8(±8.2)
	Difficulty speaking and/or discomfort with social interaction	43.2(±6.0)	44.5(±8.1)	46.8(±7.0)	49.9(±7.6)	49.8(±8.2)	46.0(±7.2)	44.7(±8.1)	45.1(±6.6)	47.1(±7.4)	45.5(±8.8)
	Pain associated with teeth or dentures	43.0(±6.0)	44.4(±8.3)	46.9(±7.0)	49.5(±7.5)	50.4(±8.0)	46.3(±7.1)	45.1(±7.8)	45.5(±6.3)	47.0(±7.4)	46.2(±8.6)

*Short Form (SF-8) domains: *GH* - General Health, *PF* – Physical Functioning, *RP* – Role Physical, *BP* – Bodily Pain, *VT* – Vitality, *SF* – Social Functioning, *MH* – Mental Health, and *RE* – Role Emotional.

**Summary scores: PCS – Physical Component Summary, MCS – Mental Component Summary.

The PCS and MCS scores at the follow-up in 2009 were closely linked to number of oral health impacts 4 years earlier (Table 3). In both age groups, we found a monotonic dose–response gradient between increasing number of oral health impacts and decreasing physical and mental summary scores. For example, an increasing number of oral health impacts were associated with declining PCS mean scores as follows: 50.5 for no oral health impact, 49.2 for one impact, 48.6 for two impacts, and 47.9 for three impacts among younger cohort members, with a similar monotonic decline among older cohort members. A similar dose–response gradient was found between an increased number of oral health impacts (0, 1, 2, and 3) and MCS mean scores in younger cohort members (43.2, 40.9, 40.3, and 38.6), with a similar monotonic decline also noted among the older members of the cohort.

**Table 3:** **Association between oral health impacts and SF**-**8 summary scores**, **Thai Cohort Study 2005**-**9**

**Age**	Oral health impacts in 2005*	2009 SF-8 Coefficients and adjusted mean scores** [95% Confidence Intervals]			
		**Physical Component Summary**		**Mental Component Summary**	
		**Coefficients**	**Adjusted means**	**Coefficients**	**Adjusted means**
<40					
	0 (reference)		50.5 [49.8-51.1]		43.2 [42.4-44.0]
	1 impact	-1.26[-1.44, -1.08]	49.2 [48.4-50.1]	-2.27 [-2.49, -2.05]	40.9 [39.9-42.0]
	2 impacts	-1.90[-2.17, -1.63]	48.6 [47.7-49.5]	-2.96 [-3.30, -2.63]	40.3 [39.1-41.4]
	3 impacts	-2.62[-3.16, -2.08]	47.9 [46.7-49.1]	-4.60 [-5.27, -3.93]	38.6 [37.1-40.1]
≥40					
	0 (reference)		51.4 [50.4-52.4]		41.5 [40.3-42.6]
	1 impact	-1.53[-1.77, -1.29]	49.8 [48.6-51.1]	-1.95 [-2.22, -1.67]	39.5 [38.1-40.9]
	2 impacts	-2.15[-2.50, -1.79]	49.2 [47.9-50.6]	-2.86 [-3.26, -2.45]	38.6 [37.1-40.2]
	3 impacts	-2.89 [-3.63, -2.15]	48.5 [46.7-50.2]	-3.74 [-4.59, -2.90]	37.7 [35.7-39.7]

*Oral health impacts include the following: difficulty chewing and/or swallowing; difficulty speaking and/or discomfort with social interaction; and pain associated with teeth or dentures.

**Adjusted for variables in Table 1 including socio-demo-geographic characteristics (age in years, sex, household monthly income, household residence), health-risk behaviors (smoking and alcohol drinking), chronic conditions (diabetes, hypertension, heart diseases), number of teeth at baseline.

## Discussion

We found consistent and significant associations between oral health impacts and general health and overall quality of life among middle-aged Thai adults four years later. Cohort members with at least one oral health impact had lower quality of life outcomes with lower SF-8 mean scores in all domains and lower physical and mental summary scores in both age groups. There were clear monotonic gradients and dose-responses between an increase in the number of oral impacts and a decrease in physical and mental health, after adjusting for potential covariates.

Our study provides empirical evidence for Thailand of the link between oral health and overall quality of life in young and middle-aged adults. This complements current literature reporting associations between oral health and quality of life and on the relationship between perceived oral and general health among adults [9,11]. Our study found oral health impacts more on mental than physical health which is consistent with findings reported among Taiwanese elderly [10]. Among oral health impacts, our data showed that cohort members with difficulty chewing/swallowing, discomfort with social setting, or pain all reported worse general health. Other European studies report similar findings, that having chewing problems and being dissatisfied with the appearance of teeth have a strong influence on self-rated general health [7,25]. Improving oral health in younger and middle-aged adults will be an important goal for developing countries, particularly to avoid worsening oral health impacts associated with ageing populations in the future [26,27].

The strengths of our study are its large number of participants, the wide array of socio-demo-geographic attributes measured, and the longitudinal follow-up, and the standard measures. We note one possible limitation is that this study is based on educated Thais and thus could underestimate the magnitude of the oral impacts in the general population. As well, the oral health questions only assessed the impact of teeth or dentures on quality of life but no details of the types and conditions of dentures were investigated. Although oral health impacts and quality of life were self-reported, these have been generally shown to correlate well with objective clinical measures of oral health status [20,21].

## Conclusions

We provide evidence for a middle-income country in the Asia-Pacific region on the strong longitudinal association between oral health and overall quality of life among Thai adults. We found monotonic dose-responses between increasing number of oral health impacts and declining physical and mental health. The evidence implies that oral health promotion to increase general public awareness and preventative oral health programs could enhance general health and quality of life among the Thai population.

## Abbreviations

OIDP: Oral Impacts on Daily Performances; MCS: Mental Component Summary; PCS: Physical Component Summary; SF: Short Form Medical Outcome Survey; TCS: Thai Cohort Study.

## Competing interests

The authors declare that they have no competing interests.

## Authors' contributions

VY and TS conceptualised, analysed and drafted the manuscript. SS and AS designed and executed the project and provided comments on the manuscript. All authors read and approved the final manuscript.

## ^†^Thai Cohort Study Team

Thailand: Jaruwan Chokhanapitak, Suttanit Hounthasarn, Suwanee Khamman, Daoruang Pandee, Suttinan Pangsap, Tippawan Prapamontol, Janya Puengson, Sam-ang Seubsman, Boonchai Somboonsook, Nintita Sripaiboonkij, Pathumvadee Somsamai, Duangkae Vilainerun, Wanee Wimonwattanaphan, Benjawan Tawatsupa, Tewarit Somkotra.

Australia: Chris Bain, Emily Banks, Cathy Banwell, Bruce Caldwell, Gordon Carmichael, Tarie Dellora, Jane Dixon, Sharon Friel, David Harley, Matthew Kelly, Tord Kjellstrom, Lynette Lim, Anthony McMichael, Tanya Mark, Adrian Sleigh, Lyndall Strazdins, Susan Jordan, Janneke Berecki-Gisolf, Roderick McClure, Vasoontara Yiengprugsawan.
